# Explaining the amount of care needed by hospitalised surgical patients: a prospective time and motion study

**DOI:** 10.1186/1472-6963-13-42

**Published:** 2013-02-04

**Authors:** Catharina J van Oostveen, Hester Vermeulen, Dirk J Gouma, Piet J Bakker, Dirk T Ubbink

**Affiliations:** 1 Department of Quality Assurance & Process Innovation, Academic Medical Centre, room A3-503, P.O box 22700, Amsterdam, DE, 1100, The Netherlands; 2 Amsterdam School of Health Professions, University of Amsterdam, P.O box 22700, Amsterdam, DE, 1100, The Netherlands; 3 Departments of Surgery, Academic Medical Centre, P.O box 22700, Amsterdam, DE 1100, The Netherlands

**Keywords:** Patient characteristics, Workload, Time and motion research, (multiple) regression analysis

## Abstract

**Background:**

Hospitals provide care for patients with a variety of diseases, co-morbidities and complications. The actual amount of care these patients need is unclear. Given the recent developments such as ageing, multi-morbidity and budgetary restraints, a practical explanatory model would avail healthcare professionals and managers in determining the demand and costs for clinical care.

**Methods:**

Six surgical wards in a Dutch university hospital participated in this prospective time and motion study. Surgeons, nurses and paramedics recorded the time spent on patient care 24/7 by means of PDAs. The investigators extracted possible determining characteristics from a previous systematic review and expert focus group. Total amount of care needed by the patients was expressed as costs involved in medical and nursing time, surgical interventions and diagnostics. Afterwards the investigators applied linear regression analysis to detect significant independent characteristics.

**Results:**

174 Surgical patients were monitored during their hospital stay. Characteristics significantly influencing the consumed amount of care were: medication during hospitalisation, complications, co-morbidity, medical specialty, age, as well as undergoing surgery and length of stay. Median costs for care were €8.446 per patient admission.

**Conclusions:**

The investigators developed a model that explains the total demand and costs of care needed for surgical patients in a university hospital. The input for this instrument can be derived from readily available data in hospital databases. This makes it a relatively easy instrument to help healthcare professionals and managers appreciate the amount of care needed on (surgical) wards and may be used to appreciate trends in time.

## Background

Given the recent societal developments such as survival to an older age, increasing multi-morbidity and stagnating growth of the working population, it is expected that the demand for medical and nursing care will increase substantially [1]. Hospitals are nowadays more and more confronted with budget cuts and accountable to substantiate their costs spent on highly specialised, top-referral care. Therefore it is important for professionals and managers to identify the factors determining the (trends in the) demand and costs for care. Surgical wards of university hospitals in particular recognize the changing demography and increasing accountability, as they rely heavily on expensive facilities like operating theatres, ICUs, and diagnostic imaging.

Demand for care is defined as the needs of individual patients in terms of the sum of (para)medical and nursing resources used [2]. One of the seminal systems to measure and classify this demand for care from nursing resources is the Therapeutic Intervention Scoring System (TISS) [3]. This instrument helps classify the workload for nurses in the intensive care unit (ICU) by registering and weighing therapeutic nursing interventions. On general hospital wards, similar instruments are used as Patient Classification Systems (PCS). These instruments rely on subjective and clinical observations by nurses, give information about nursing care already given, and help with the staffing of nursing wards [4]. These instruments, however, cannot predict the demand for care, particularly from doctors and paramedics, and are not based on objective measures, such as patient characteristics.

Few studies have investigated objective influencing factors for nursing workload [2,5-14]. Although these studies applied the demand for care as a reference standard, they used different definitions for this entity [15,16]. This led to invalid and unreliable PCSs and systematic under- and overestimation of the demand for care, while the explored characteristics per se were poorly associated.

The investigators therefore aimed to develop an explanatory model, based on readily available clinical patient characteristics from hospital databases for the use of (para)medical and nursing resources by surgical patients. A practical explanatory model would avail healthcare professionals and managers in determining the demand and costs for clinical care and use this information for policy making, i.e., budget planning.

## Methods

The conduct and description of this study was done according to the Suggested Time And Motion Procedures (STAMP) checklist [17].

### Setting

Six general surgical wards in a university hospital in The Netherlands contributed to this study. These 24-bed wards provide standard and specialty surgical care, i.e. general, vascular, plastic, orthopaedic, and trauma surgery. On each ward approximately 30 nurses, three auxiliary nurses, one resident, two surgeons, one physical therapist, one social worker, and one dietician were involved during the study.

### Design

In this prospective time and motion study medical, nursing and paramedical personnel continuously (24/7) recorded the patients’ care process during admission.

The investigators recorded data on diagnostic and surgical procedures, intensive care stay, total length of hospital stay, and time spent on patient care by all caregivers (doctors, nurses and paramedics). Time recordings comprised direct patient contact or indirect care (i.e. patient-related telephone calls, planning and administrative activities, inter-professional consultations, multidisciplinary meetings, etc.). These data were used as reference standard to develop the desired explanatory model.

### Potentially predicting patient characteristics

A set of 17 potentially predictive characteristics (Table 1) was defined based on suggestions made by a local expert panel (consisting of head nurses, nursing managers and clinicians), and a systematic literature review not yet published. These characteristics could be extracted from the medical and nursing files and hospital databases and therefore did not need any additional registration effort. Co-morbidities were counted if requiring treatment with drugs or medical devices (e.g. prosthesis).

**Table 1:** Potentially predictive patient characteristic

**Characteristic**	**Range of possible values**
Surgical intervention	0= yes, 1= no
Date of birth	0 to ∞
Gender	0= woman, 1= man
Number of co-morbidities	0 to ∞
Number of complications	0 to ∞
ASA-classification	1, 2, 3 or 4
BMI at admission	0 to ∞
Nutritional status (weight loss in past 6 mo.)	0 to ∞ kg
Delirium during hospitalisation	0= no, 1= yes
Pressure ulcer during hospitalisation	0= no pressure ulcers, or grade 1 through 4 ulcers
Isolated care during hospitalisation	0= no, 1= barrier, 2= strict isolation
Survival during hospitalisation	0= yes, 1= no
Number of different medications during hospitalisation	0 to ∞
Admission type	0= home, 1= emergency
Discharge type	0= home, 1= other
Length of hospital stay	number of days
Surgical specialty	TRAUMA Trauma surgery
	URO Urology
	SHORT Short Stay surgery
	ORTHO Orthopaedics
	ABDO G-I surgery
	PLAST Plastic surgery
	VASC Vascular surgery
	ORAL Oral and Maxillofacial surgery

Patient characteristics were collected and checked by two investigators independently during admission and after discharge from the hospital. Only co-morbidities requiring medication or a medical device were recorded. Missing data were retrieved from medical and nursing files or asked directly from the patients during their hospitalisation or, after discharge, by phone.

### Patient sample

To define a patient sample representative for those regularly admitted to surgical wards, the investigators used the latest available update of the national medical registry (LMR) of admission diagnoses. To develop an explanatory model with up to 17 predefined characteristics, the investigators decided to collect a sample of at least 170 patients in a three-month period. The numbers of patients with a certain diagnosis to be included was commensurate with the ranking based on a top-12 of admission diagnoses for each ward (Table 2). Patient inclusion stopped when a sufficient number of patients with these diagnoses was reached.

**Table 2:** Patient samples per surgical specialty

**Specialty**	**Top 12 admission diagnoses**	**Estimated diagnosis incidence**	**Planned patient inclusion**	**Realised patient inclusion**
URO	**Diseases of the genitourinary system**	406 (50.1%)	23	(47.8%) 11
SHORT	**Diseases of the digestive system**	113 (14%)	7	(57.1%) 4
	**Neoplasms**	301 (37.2%)	17	(82.4%) 14
	*Additional inclusions*			4
	**Total**	**810 (100%)**	**47 (100%)**	**(71.7%) 33 (19%)**
VASC	**Diseases of the circulatory system**	208 (55.8%)	12	(66.7%) 8
PLAST	**Diseases of the skin and subcutaneous tissue**	24 (6.4%)	1	(100%) 1
	**Diseases of the genitourinary system**	28 (7.5%)	2	(100%) 2
	**Diseases of the musculoskeletal system and connective tissue**	54 (14.5%)	3	(66.7%) 2
	**Factors influencing health status and contact with health services**	59 (15.8)	4	(75%) 3
	*Additional inclusions*			7
	**Total**	**373 (100%)**	**22 (100%)**	**(100%) 23 (13.2%)**
ABDO	**Neoplasms**	204 (63%)	12	(125%) 15
	**Diseases of the digestive system**	106 (32.7%)	6	(133.3%) 8
	**Factors influencing health status and contact with health services**	14 (4.3%)	1	(200%) 2
	*Additional inclusions*			2
	**Total**	**324 (100%)**	**19 (100%)**	**(142.1%) 27 (15.5%)**
ABDO	**Neoplasms**	153 (58.8%)	10	(160%) 16
ORAL	**Diseases of the digestive system**	83 (32%)	5	(300%) 15
	**Injury, poisoning and certain other consequences of external causes**	24 (9.2%)	1	(300%) 3
	*Additional inclusions*			4
	**Total**	**260 (100%)**	**16 (100%)**	**(237.5%)38 (21.8%)**
TRAUMA	**Injury, poisoning and certain other consequences of external causes**	311 (88.1%)	18	(66.7%) 12
	**Diseases of the musculoskeletal system and connective tissue**	42 (11.9%)	2	(150%) 3
	*Additional inclusions*			9
	**Total**	**353 (100%)**	**20 (100%)**	**(120%) 24 (13.8%)**
ORTHO	**Injury, poisoning and certain other consequences of external causes**	124 (27.2%)	7	(57%) 4
	**Diseases of the musculoskeletal system and connective tissue**	286 (62.7%)	16	(113%) 18
	**Neoplasms**	46 (10.1%)	3	(133.3%) 4
	*Additional inclusions*			3
	**Total**	**456 (100%)**	**26 (100%)**	**(111.5%) 29 (16.7%)**
	***Overall total***		**150 (100%)**	**(116%) 174 (100%)**

### Study conduct

**Figure 1:**
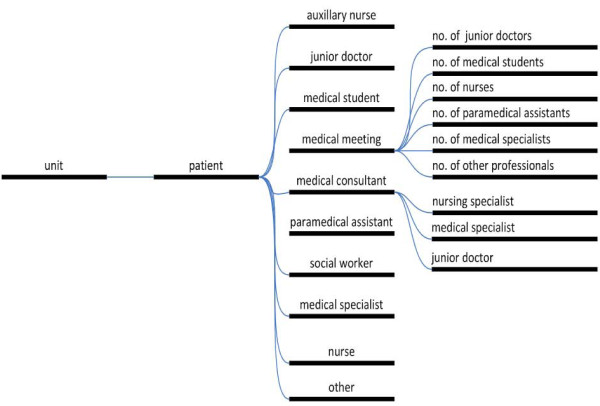
Menu structure of Personal Digital Assistants.

For continuous time and motion research the investigators used Personal Digital Assistants (PDAs) (PalmOne Tungsten E2; Palm Inc., Sunnyvale, CA, USA). A dedicated software program was developed for this purpose (I-V-O: Web development, scripting, hosting & consultancy, Alkmaar, The Netherlands). This allowed recording of the date, duration of direct and indirect time spent per patient, type of professionals involved, and wards and patients involved (Figure 1). Data thus collected were downloaded daily from the PDAs to a central computer database. The PDAs were distributed during every shift to the professionals involved on each ward. Consulting professionals visiting an included patient made use of an additional PDA placed at the patients’ bedside. All professionals contributing to the study were informed about its purpose and the use of the PDAs by instructive posters and meetings.

Eight investigators distributed the PDAs and were available for support seven days a week from 7AM to midnight. Recording errors were as much as possible recognised directly by means of a logbook and corrected and evaluated afterwards. Recorded time data were checked and analysed twice a week for exceptional and missing values. Such recordings were replaced by an average, based on similar situations in the same patient. To check the reliability of the data the investigators frequently asked, and randomly shadowed, the professionals involved regarding their recording behaviour.

### Time and motion method

To obtain a single measure for demand for care, information about wards, patient characteristics, professionals involved, date, time, and duration of care were converted into the costs involved. Standard costs for wages of the various professionals were used for day, evening, night, and weekend shifts, whenever applicable. Costs of surgical interventions were based on the gross time needed for the surgical procedure and the associated salary costs of the professionals present. The costs of diagnostic procedures and ICU and recovery stays were added to arrive at the total costs of the demand for care for each patient during their admission period. The total costs were used as the dependent variable in the explanatory model.

To account for the possible influence of the availability of resources on the amount of care given, the investigators also observed the relation between available Full Time Equivalents (FTE) and bed occupancy rates per ward.

### Statistical analysis

Data were imported into the Statistical Package for the Social Sciences v. 16 (SPSS Inc., Chicago, IL, US). Categorical data are presented as proportions. Continuous variables are summarised as means with standard deviations.

After exploration of the association between the various characteristics and the costs of the demand for care in a univariable analysis, significant predictive characteristics were detected in a multiple backward linear regression analysis. Additionally, each non-significant factor was added one by one to the model found by the multiple backward analysis to check whether they contributed significantly to the model.

To distinguish patient characteristics and organisational factors, we analysed these in different models. For all analyses the significance level was set at P<0.05. B-values were calculated with their 95% confidence intervals. Log-transformation of the dependent variable total costs of demand for care was performed because of its non-normal distribution.

### Ethical issues

Our local medical ethics review board (Academic Medical Centre, Amsterdam, The Netherlands) approved the study but waived the need for written informed consent, as the study had no effect on the patient’s treatment or psychological wellbeing. Yet, all included patients received an explanation about the study and gave verbal consent.

## Results

From February to April 2010, 174 consecutive patients were included, both elective and emergency admissions. One patient declined participation in the study. Demographics of included patients are summarised in Table 3.

**Table 3:** Univariable and multivariable linear regression analysis of possible predictive characteristics

				**Uni-variate**			**Multi-variate**		
**Characteristic**	**N (%)**	**Mean (SD)**	**Range**	**Estimate (B)**	**95% CI**	**P-value**	**Estimate (B)**	**95% CI**	**P-value**
Age		57.2 (16.6)	19-87	0.004	0.001–0.007	0.004	0.002	0.000–0.005	0.072
Surgical Intervention performed	167 (96)			0.594	0.351–0.837	<0.001	0.466	0.288–0.643	<0.001
Gender (males)	99 (56.9)			-0.015	–0.118–0.870	0.767			
Number of co-morbidities		1.47 (1.68)	0-9	0.000	–0.031–0.030	0.978	–0.038	–0.064–0.012	0.005
Number of complications		0.21 (0.60)	0-4	0.221	0.144–0.299	<0.001	0.072	0.005–0.139	0.036
ASA-class									
1	41 (26.8)			RC					
2	89 (58.17)			0.168,	0.057– 0.279	0.003			
				RC					
3	23 (15.03)			0.234,	0.081–0.387	0.003			
				0.067	–0.071–0.204	0.339			
BMI at admission		26.43 (5.37)	17.2-53.6	–0.006	–0.015–0.003	0.189			
Nutritional status		2.28 (5.78)	0-50	0.018	0.010–0.026	<0.001			
Delirium during hospitalisation	3 (0.7)								
Pressure ulcer acquired during hospitalisation									
grade 1	0								
grade 2	1 (0.6)								
grade 3	1 (0.6)								
grade 4	0								
Isolation									
barrier	2 (1.15)								
strict isolation	0								
Survival	174 (100)								
Number of medications during hospitalisation		8.51 (5.07)	0-26	0.031	0.022–0.040	<0.001	0.013	0.004–0.023	0.007
Admission type				–0.210	–0.360–0.061	0.006			
home	152 (87.36)								
emergency	22 (12.64)								
Discharge type									
home	163 (93.7)								
other	11 (6.32)								
Length of Stay		8.11 (6.85)	1-45	0.034	0.028–0.039	<0.001	0.032	0.027–0.037	<0.001
Surgical specialty									
TRAUMA	4 (2.3)			RC					
URO	21 (12.07)			0.776	0.511–1.042	<0.001	0.760	0.500–1.021	<0.001
ORTHO	49 (28.16)			0.758	0.505–1.012	<0.001	0.706	0.461–0.950	<0.001
ABDO	55 (31.06)			1.152	0.900–1.405	<0.001	1.005	0.755–1.255	<0.001
SHORT	14 (8.05)			0.644	0.368–0.920	<0.001	0.623	0.350–0.896	<0.001
PLAST	12 (6.9)			0.622	0.381–0.943	<0.001	0.610	0.339–0.882	<0.001
VASC	11 (6.32)			0.786	0.502–1.071	<0.001	0.738	0.456–1.020	<0.001
ORAL	8 (4.6)			0.679	0.380–0.977	<0.001	0.664	0.383–0.946	<0.001

RC=reference category.

**Figure 2:**
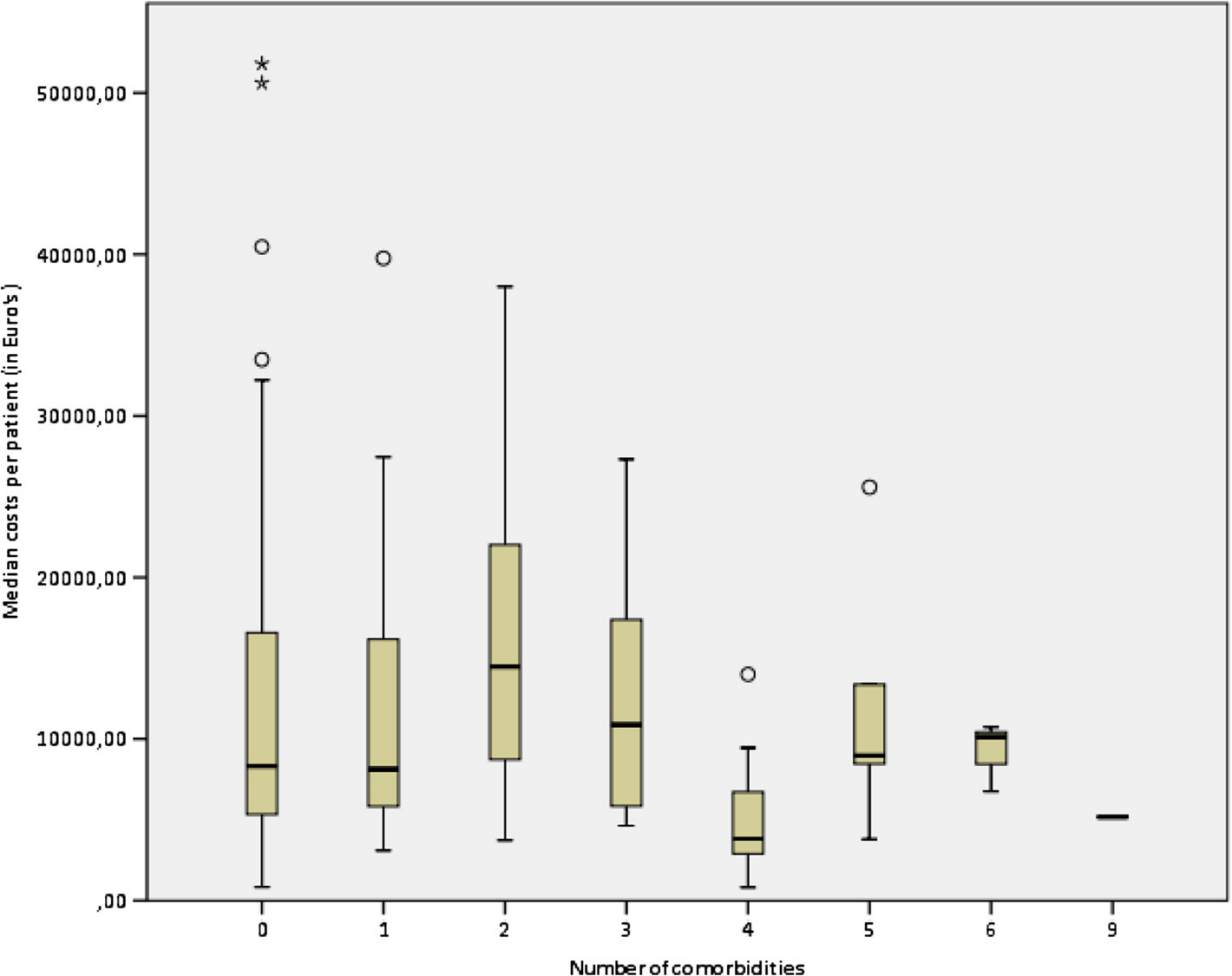
Box plots of median costs per patients of the demand for care per number of co-morbidities.

**Figure 3:**
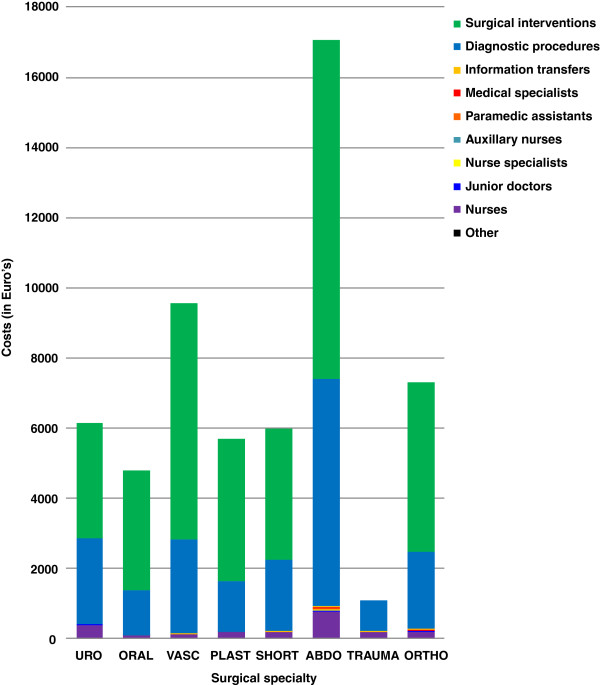
Median costs per patient of the demand for care per surgical specialty.

Median total costs of the demand for care per patient were €8,446 and varied from €815 for trauma patients to €82,780 for G-I surgical patients (Figure 2). Surgical and diagnostic interventions contributed most to these costs. Nursing costs formed the largest part (76%) of the personnel expenses; €308, vs. physicians €56, and paramedics €2.70 per patient, excluding the personnel costs for the surgical intervention. Median costs for surgical interventions were €5,286 (range: €0 – €21,111). Median costs for diagnostic procedures were €2,699 and varied from €372 to €74,567 (Figure 3).

Median total costs of the demand for care per patient were €8,446 and varied from €815 for trauma patients to €82,780 for G-I surgical patients (Figure 2). Surgical and diagnostic interventions contributed most to these costs. Nursing costs formed the largest part (76%) of the personnel expenses; €308, vs. physicians €56, and paramedics €2.70 per patient, excluding the personnel costs for the surgical intervention. Median costs for surgical interventions were €5,286 (range: €0 – €21,111). Median costs for diagnostic procedures were €2,699 and varied from €372 to €74,567 (Figure 3).

In the univariable analysis, age, number of complications, ASA-class, nutritional status, admission type, number of medications during hospitalisation, and surgical specialty were significantly associated with the costs of demand for care (Table 3), as opposed to gender, number of co-morbidities, and BMI. Delirium and isolation during hospitalisation, pressure ulcers, admission type, and mortality did not contribute significantly, likely because they occurred rarely. A total of 153 valid cases from the initial 174, i.e. without any missing values, could be used to complete the multivariable regression analysis. Although not significant in the univariable analysis, number of co-morbidities was also used in the multivariate analysis because of the allegedly high clinical relevance of this characteristic.

The best model of patient characteristics to predict the total costs of the demand for care contained the number of medications during hospitalisation, number of co-morbidities, number of complications, age and surgical specialty. This model explained 56.2% of the variance in the demand for care in terms of costs. The set of dummies for surgical speciality effectuate 49% of this variance. Total costs increased with 18% per additional complication (95% CI 1 to 38%; p=0.036), while an additional medication caused a 3% increase in costs (95% CI 1 to 5%; p=0.007). Per increasing year in age the costs increased with 0.5%, but this was not statistically significant (95% CI 0 to 1%; p=0.072). Unexpectedly, an additional co-morbid condition lowered the costs with 9% (95% CI -16 to -3%; p=0.005; Figure 2). In addition, in a separate model of organisational factors surgical intervention and length of hospital stay were also found to be significant factors of total costs of care. This model explained 54% of the demand for care. Undergoing a surgical procedure nearly tripled total costs (292%; 95% CI 194 to 440%; p<0.001), while an extra day of hospitalisation increased the costs with 8% (95% CI 6 to 9%; p<0.001).

Bed occupation as proportion of the total number of available beds varied among wards from 53.2 to 70.5%, while the percentage of optimum staffing in FTEs per ward ranged from 93.4 to 96.6% (Table 4). The investigators did not find any relation between a higher FTE occupancy or lower bed occupancy and more time spent on care. Hence, we could not detect a substantial influence of the availability of resources on demand for care.

**Table 4:** Bed occupation and available FTEs during data collection

**Unit**	**Bed occupation %**^**1,2**^	**FTE**^**2,3**^	**Time**^**4**^
Short stay & Urology	60.29	95.08%	32:19:06
Vascular and Plastic surgery	56.63	93.62%	29:53:37
G-I surgery	65.86	94.85%	67:44:50
G-I surgery and Oral & Maxillofacial surgery	70.53	93.44%	63:12:02
Trauma surgery	53.20	96.58%	30:03:24
Orthopaedic surgery	62.09	93.55%	35:29:41

1: Bed occupation (realised/available beds) during study period.

2: Mean of February and March 2010.

3: 100% (optimum personnel staffing) minus absence.

4: Mean total time spent per patient during hospitalisation.

From our random checks of the completeness of data recordings the investigators appreciated that nurses recorded 59 to 96% of their times spent per patient. Physicians stated a registration of between 45 to 100% of their activities.

## Discussion

A model was developed to explain the demand for care based on readily available patient characteristics. Number of medications during hospitalisation, number of co-morbidities, number of complications, age, surgical specialty, as well as undergoing a surgical intervention and length of stay significantly contributed to an increased demand for care. It is likely that these results are generalizable to other specialties because these are blanket factors, applicable to a broad patient population.

No significant associations were found between the patient’s ASA class, nutritional status, admission type and their demand for care. This is partially in agreement with the results from other investigators [10], a weak but significant correlation (r=0.35 p<0.0001) between admission type and nursing workload. For ASA class and nutritional status, no comparable evidence is available. ASA class appeared to be a promising influencing factor in the univariable analysis, but was found not significant in the multivariable analysis. Probably too few patients belonged to ASA class 3, because we found significant associations between ASA classes 1 and 2, and between 1 and 3, but not between classes 2 and 3. Also delirium, pressure ulcers, patient isolation, and in-hospital mortality were not significantly associated with demand for care. This is likely because their incidence was quite low in our study, but not unusual for these wards. Furthermore, these factors are less useful as factors predicting the demand for care because they occur during hospitalisation and are not known beforehand. If they would contribute significantly to the model, they can still be useful as a managerial tool to monitor amount of care on a more aggregate level on wards to detect trends in time as to patients’ demand for care.

Some nursing care models have found the case-mix groups (CMG) or Diagnosis Related Groups (DRGs) to be explanatory factors for the demand for nursing care [6,9,11-13]. In this study the investigators categorised the medical diagnoses at a more abstract level, i.e., surgical specialty, because of the large variety in diagnoses present. This specialty appeared relevant as it showed to be an important significant factor, explaining 49% of the variance in the demand for care in terms of costs.

The number of complications during hospitalisation also had a large influence on the demand for care. This number is likely to be related to co-morbidity and medication. Therefore, this number seems a sensitive indicator for the complexity of care and the following demand for care. Complexity is an important concept in research as to the demand for nursing care [12,14]. In the nursing realm, complexity has been measured by parameters like severity of illness [10] nature of nursing tasks [12,18] and nursing diagnoses [9,12]. These variables had similar predicting values. The impact of complications on the demand for care was mainly due to the costs for diagnostic or therapeutic interventions, such as (redo) surgery to treat complications, and mostly occurred in patients undergoing gastro-intestinal surgery. This may be exemplary for the tertiary referral hospital in which this study was conducted.

The number of medications used during hospitalisation had less influence on the demand for care. No comparable evidence is available but this limited influence is possibly caused by the fact that medication is principally given to cure, and therefore associated with an increase in the demand for care. Also ‘age’ had less influence on the demand for care. This parameter nearly reached statistical significance (P=0.072) in the multivariable model and was added because of its clinical relevance. Such poor associations were also found by other researchers [8,9,12].

The negative association found between co-morbidity and demand for care may be because the severity of the various co-morbidities was not weighed in this study. Less severe co-morbidities may have been managed through medication, while patients with more severe co-morbidities were less likely to undergo surgery. This is confirmed by the study of Gijsen et al. [19]. They proposed the Charlson Comorbidity Index (CCI) to operationalise the severity of the co-morbidity.

Also, undergoing a surgical intervention and length of hospital stay were significant factors associated with the demand for care. This seems obvious, given the additional costs of surgery and of each extra day spent in the hospital. Previous studies have shown this is likely to be related to the severity of the patient’s illness and therefore their demand for care [2,10,12].

Some limitations of this study should be discussed. First, the investigators calculated and modelled the care the patients received, which may not be commensurate with what they needed. We did check that the results of our study represented demand for care rather than the mere usage of personnel and resources. The delivered care was independent of bed occupancy and available personnel. This suggests that indeed the demand for care was measured instead of offered resources. In retrospect, the investigators might also have appreciated whether the care given had met the patient’s expectations and had cured or relieved their disorder.

Second, the investigators used a diversity of input, structure, process, and outcome variables in the model. As mentioned earlier, variables occurring during hospitalisation are unknown beforehand and therefore not useful as predictive factors. It seems plausible to use input variables for the explanatory model and use process and structure variables as specialty-specific or centre-specific characteristics, e.g. undergoing a surgical intervention, level of education [7,8], or organisational factors [12,13], in an additional model. Furthermore, the success of the care given could also have been estimated, e.g. by measuring outcome variables as the number of complications or readmissions within 30 days after dismissal or by appreciating the quality of care [8]. This was beyond the possibilities of the present study, but will be incorporated in a recently started follow-up study among Dutch top-clinical hospitals.

Third, the investigators took for this study an innovative approach to measure the demand for care by time and motion research. This method was performed with rigour to collect data on individual patient contacts by professionals. Otherwise, continuous time and motion research provides precise results only if the professionals involved are willing to accurately record the time spent. The investigators found under-recording of time data, predominantly among doctors, resulting in an under-reporting of the total costs involved. Although this will have weakened the power of our model to predict demand for care, there was no reason to suspect selective under-recording that would have influenced the ability to detect predictive characteristics. It may explain, however, that the demand for care in our model appeared determined by the costs of the surgical and diagnostic interventions rather than the costs of personnel outside the operating theatre. As the investigators could not incorporate all costs at the same level of detail (e.g. overhead cost on wards or surgical interventions were not taken into account), a representative estimate was used of the costs for (para)medical and nursing care during admission. However, the overhead costs are likely to be proportional to the personnel costs we measured and therefore not influencing the outcome of our model.

Fourth, by expressing the demand for care as costs, the contribution of unpaid medical trainees to the patient care was not taken into account, although they deliver a substantial contribution to patient care in university clinics and affiliated hospitals. In addition, costs for overhead, patient transport, medication, material costs for surgical procedures in the operating theatre or on the nursing ward were not taken into account, while costs for ICU- and recovery stays were entered as fixed costs. Finally, no additional charges were included for surgical interventions during weekends, evenings and nights. Further detailing of these costs was beyond our possibilities but it is doubtful whether this would have had a major impact on the general outcome of our study.

## Conclusion

A practical model was developed to explain the total demand and costs of care for surgical patients in a university hospital. The input for this model, age, number of co-morbidities, number of medications during hospitalisation, number of complications, surgical specialty, and length of hospital stay, can be derived from readily available data in hospital databases. The time and motion approach to estimating costs potentially provides an accurate assessment of the demand for care. This approach can be applied more broadly to the same ends.

It is worthwhile to explore this model in different populations and healthcare organisations. The results needs to be further explored, but can combined with population projections potential allow healthcare professionals and managers in policy making, i.e. informed planning and budgeting.

## Competing interest

The authors declare that they have no competing interests.

## Authors’ contributions

CO, DU and HV were responsible for the conception; CO and DU designed the study; CO, DU and HV were responsible for data collection; CO and DU analysed the data; CO and wrote the article; CO had primary responsibility for final content; HV, DG and PB were responsible for editing the article. All authors read and approved the final manuscript.

## Pre-publication history

The pre-publication history for this paper can be accessed here:

http://www.biomedcentral.com/1472-6963/13/42/prepub
